# Drivers of behaviors: How do city pilots shape residential energy-related emissions through perceptions?

**DOI:** 10.3389/fpsyg.2023.1127227

**Published:** 2023-03-09

**Authors:** Hua Xing, Xiangyang Li

**Affiliations:** School of Government, Central University of Finance and Economics, Beijing, China

**Keywords:** low carbon city pilots, residential perceptions, energy consumption, theory of planned behavior, difference in difference

## Abstract

Residential energy consumption, as a major source of emissions in cities, is also a policy priority for the construction of low-carbon cities. The occurrence of residential energy saving and emissions mitigation behaviors is closely related to low-carbon perceptions. Against this background, cities make efforts to shape residential low-carbon perceptions. In order to investigate residential energy consumption and carbon emissions, this study takes low-carbon city pilots as the policy context and establishes the difference-in-difference model on Chinese prefecture-level cities. Theory of planned behavior is utilized to analyze the influence mechanism of residential low-carbon perceptions. Results indicated that (1) low-carbon city pilots can decrease residential energy-related emissions and pass a variety of robustness tests. Multiple pilot eligibility and policy lag would reinforce policy effects. (2) Mechanism analysis shows that low-carbon city pilots can strengthen residential behavioral attitudes, establish subjective norms, and adjust perceived behavioral control. All three mechanisms together shape residential low-carbon perceptions, which consequently promote energy-related emissions mitigation behaviors. (3) Due to differences in geographic location and city size, there is heterogeneity for the policy effects of low-carbon city pilots. For the future research, it is necessary to expand the scope of residential energy-related emissions, find out the potential influencing factors, and track the policy effects in long-term.

## Highlights

- Determining whether pilot cities would adjust residential low-carbon behaviors.
- The causal chain from policy to low-carbon perceptions to behaviors clarified.
- TPB as the theoretical framework analyzed the influence mechanisms.
- Heterogeneity analysis helps cities with low-carbon development.

## 1. Introduction

Residential energy consumption is one of the major sources of carbon emissions in cities. In developed countries, 70–90% population lives in cities (Miao, 2017). Subsequently, cities contribute 80% of global energy consumption while generating 60% of greenhouse gas (GHG) emissions (Harris et al., 2020). In this regard, the share of residential energy-related emissions in the United Kingdom and the United States are 74 and 80%, respectively. In China, residential energy consumption accounted for about 30–40% and the ratio will continuously increase in the future (Wang and Yang, 2014; Li et al., 2019). Residential energy consumption and carbon reduction are one of the five major fields within the policy contents about low-carbon city pilots (LCCP). While implementing LCCP, cities decrease residential energy-related emissions by disseminating scientific information to residents, propagating low-carbon ideas, organizing energy-saving activities, stimulating green behaviors, improving infrastructure, and providing urban services (Li et al., 2018; Westman and Broto, 2018). Thus, energy consumption behaviors and low-carbon perceptions of residents in LCCP policy contexts should be the research perspective in focus.

At present, there is a lack of attention to residential low-carbon perceptions and energy consumption behaviors under LCCP. A few studies concentrate on the policy analysis of LCCP, those studies analyze the aspects of policy contents (Li et al., 2018; Wang et al., 2018), policy instruments (Ma et al., 2021), and policy innovations (Guo et al., 2021; Song et al., 2021). Other studies are concerned with the policy effect analysis of LCCP on carbon emissions mitigation in cities (Yu and Zhang, 2021; Huo et al., 2022) and corporates (Chen et al., 2021). Moreover, several studies have analyzed factors that influence residential low-carbon perceptions and behaviors, such as psychological distance (Wang et al., 2019; Jiang et al., 2020), political orientation (Luo and Zhao, 2019; Gregersen et al., 2020), climate experience (Yang et al., 2021), education level (Wang and Zhou, 2020), social norms (Sörqvist and Langeborg, 2019), and local environment (Cianconi et al., 2020; Zhang et al., 2020). However, there is no research on residential low-carbon perceptions and energy consumption behaviors taking LCCP as the policy context.

To fully understand the policy effects of LCCP, this study focuses on carbon emissions due to residential energy consumption behaviors. The purpose of this study is to investigate whether LCCP would decrease residential energy-related emissions and what influencing mechanisms affect residential low-carbon perceptions and energy consumption behaviors. First, the difference-in-difference (DID) model on residential energy-related emissions is constructed according to LCCP, a quasi-natural experiment. Second, an inventory of residential carbon emissions in cities is constructed depending on their household living energy consumption types. Finally, the theory of planned behavior (TPB) serves as a bridge to analyze perceptions and behaviors, providing the theoretical basis for further understanding of how LCCP influences residential low-carbon perceptions and subsequently drives carbon emissions mitigation behaviors.

Compared with previous literature, the potential contributions of this study are as follows: (1) Examining the changes in residential energy consumption and carbon emissions caused by LCCP, which provides a new research perspective for the evaluation of policy effects. (2) Focusing on how LCCP shaped residential low-carbon perceptions, subsequently driving energy-saving behaviors, it clearly reveals the causal chain of residential energy-related emissions. (3) Introducing TPB as a theoretical framework facilitates illustrating the influence mechanisms of LCCP on residential perceptions from attitude, norm, and perception perspectives, which provides stronger explanatory power for policy effects. (4) Distinguishing the heterogeneity of LCCP according to geographic location and city size helps cities to promote the construction of low-carbon cities with their own characteristics.

The rest of this study includes: Section 2 is the theoretical analysis, covering the literature review and theoretical hypotheses. Section 3 contains methodology and data, describing the DID model and data sources. Section 4 is the empirical results to describe whether LCCP can decrease residential energy-related emissions, with a series of robustness tests. In section 5, further analysis is to explore the influencing mechanism and heterogeneity on how LCCP can change energy-related emissions by shaping residential low-carbon perceptions. Section 6 presents the main conclusions and policy recommendations.

## 2. Theoretical analysis

### 2.1. Literature review

Existing researches relevant to residential energy consumption behaviors and low carbon perceptions under LCCP are as follows.

First, policy contents and effects of LCCP. China has implemented three batches of LCCP to decrease energy consumption and carbon emissions in cities. Scholars have summarized policy measures in five areas: planning design, supporting policies, emissions inventories, low-carbon industries, and residential energy consumption (Wang et al., 2018; Westman and Broto, 2018; Peng and Bai, 2021). Many pilot cities have launched initiatives to decrease residential energy-related emissions, such as popularizing scientific knowledge, promoting low-carbon lifestyles, conducting campus education, and organizing energy-saving activities (Zhao et al., 2016; Li et al., 2018). Evaluation of policy effects in LCCP has been carried out by establishing indicator systems (Peng and Deng, 2021), factor decomposition (Qu and Liu, 2017; Cai et al., 2018), and causal inference (Hong et al., 2021; Shen et al., 2021; Liu et al., 2022). Among them, many scholars have considered LCCP as a quasi-natural experiment and adopted the DID model to examine causal relationships between LCCP and urban emissions (Huo et al., 2022), green growth (Cheng et al., 2019), eco-efficiency (Song et al., 2020), and corporate emissions (Chen et al., 2021). Despite being one of the major policy contents in LCCP, there are no researches to analyze the policy effects on residential energy consumption and carbon emissions.

Second, studies on accounting and influencing factors of residential energy-related emissions. Some works have calculated residential energy-related emissions by means of surveys (Li et al., 2019), input-output method (Fan et al., 2012; Xia et al., 2019), or consumer lifestyle approach (Chen et al., 2019). Nevertheless, these results are weak in accuracy and authenticity. Other researches measure residential energy consumption and carbon emissions from household living, such as appliances (Miao, 2017), housing (Ma et al., 2022a), cooking (Zhang et al., 2017), and transportation (Lin and Du, 2015). In addition, several works focusing on carbon emissions from heating, lighting, appliances, and cooling in commercial buildings (Xiang et al., 2022) are also very enlightening. The research scope ranges from urban clusters (Ma et al., 2022b) to national (Zhang et al., 2022a,b) to global (Xiang et al., 2022). Meanwhile, methods to identify the influencing factors of residential energy-related emissions include the log-mean divisia index (LMDI) method (Ma et al., 2022a), stochastic impacts by regression on population, affluence, and technology (STIRPAT) (Miao, 2017), latent dirichlet allocation (Wu et al., 2021), and driving force analysis (Shen et al., 2018). However, these methods are mainly based on conditions, such as economic development, population scale, household income, and technological progress, and have not provided deeper insight into the interrelationship between residential energy-related emissions and local policies.

Third, surveying and profiling for residential low-carbon perceptions and behaviors. To better understand the relationship between low-carbon perceptions and behaviors of residents, many scholars and institutions have conducted surveys and interviews since the 1990s (Wang and Zhou, 2020). Although complete information could not be obtained, survey research is increasingly deepening the understanding of residential low-carbon perceptions and behaviors (Yang et al., 2021). Researchers measured subjective factors such as psychological distance (Wang et al., 2019), emotional characteristics (Lehman et al., 2019; Galway and Beery, 2022), political orientation (Luo and Zhao, 2019; Gregersen et al., 2020), education level (Wang and Zhou, 2020), and social norms (Sörqvist and Langeborg, 2019). External environmental factors include climate experience (Bø and Wolff, 2020; Sambrook et al., 2021; Yang et al., 2021), local conditions (Cianconi et al., 2020; Zhang et al., 2020), and environmental management (Marshall et al., 2019), and so on. Upon these, classical theories on perception and behavior have grown, such as the value-belief-norm theory (Kiatkawsin and Han, 2017; Sarkis, 2017), attitudinal-behavior-circumstance (ABC) theory (Ding et al., 2018), and TPB (Huang and Ge, 2019). TPB underlines that intentions determine individual behaviors through behavioral attitudes, subjective norms, and perceived behavioral control (Tian et al., 2022). TPB builds a bridge between the perceptions and behaviors of residents with more explanatory power. Therefore, this study utilizes TPB as a theoretical framework to explain how LCCP can shape residential low-carbon perceptions and subsequently drive mitigation behaviors.

### 2.2. Theoretical hypothesis

Promoting low-carbon lifestyles and mitigating energy consumption are indispensable policy contents of LCCP. As a comprehensive policy, it contains design planning, supporting policies, monitoring systems, industrial greening, and low-carbon living (Li et al., 2018; Westman and Broto, 2018; Peng and Bai, 2021). Overall, LCCP significantly decreased energy consumption and carbon emissions in pilot cities, and by a bigger margin than in other cities (Wang et al., 2015). For residential energy-related emissions, pilot cities also explore various initiatives to carry out, such as supplying clean energy, green transportation systems, improving building energy efficiency, and popularizing low-carbon ideas (Zhao et al., 2016; Ma et al., 2021). At the same time, residents have reacted positively to low-carbon policies by supporting emissions mitigation policies and taking low-carbon behaviors (Tian et al., 2022). Therefore, as policy priorities of LCCP, residential energy consumption and carbon emissions would be adjusted by pilot policies.

Hypothesis 1: Low-carbon city pilots would decrease residential energy-related emissions.

To better understand the influence mechanisms of LCCP on residential energy-related emissions, this study introduces TPB to diagnose the relationship between residential low-carbon perceptions and behaviors. TPB emphasizes that residential behaviors are derived from the combination of three perceptions: behavioral attitudes, subjective norms, and perceived behavioral control (Ding et al., 2018). This study utilizes TPB as a theoretical framework to investigate how LCCP influences residential behavioral attitudes, subjective norms, and perceived behavioral control and subsequently drives residential energy-related mitigation emissions behaviors.

First, behavioral attitude is a personal positive or negative affective tendency toward particular behaviors. It is influenced by the combination of rational evaluations and likelihood assessments of behavioral outcomes. Attitudes have remarkable effects on low-carbon behavioral intentions and realistic behaviors, and other related attitudes (attitudes toward science and climate) also influence residential low-carbon behaviors (Ding et al., 2018). Residents in cities are willing to buy environmental-friendly products, even if they pay more, as well as strongly support climate policies (Yang et al., 2014; Huang and Ge, 2019; Tian et al., 2022). Especially in some pilot cities, residential behavioral attitudes are influenced are reinforced by disclosing environmental information, establishing low-carbon museums, and encouraging waste recycling (Zhao et al., 2016; Ma et al., 2021). Therefore, LCCP seeks to influence residential behavioral attitudes and in turn motivate emissions control behaviors.

Hypothesis 2: Low-carbon city pilots would strengthen the behavioral attitude of residents to promote emissions mitigation behaviors.

Next, subjective norms are external pressures that individuals sense. These pressures generated by other individuals or society make individuals form their own judgments about whether they should do or not. Individual low-carbon perceptions and behaviors would be influenced by psychological distance (Wang et al., 2019), emotional characteristics (Lehman et al., 2019; Galway and Beery, 2022), political orientation (Luo and Zhao, 2019; Gregersen et al., 2020), education level (Wang and Zhou, 2020), and social norms (Sörqvist and Langeborg, 2019). In China, where the government dominates the narrative and policy agenda of every societal challenge, concerns about climate change are focused on the environment and health. As a result, climate skepticism is not prominent, and education has a strong influence on the subjective factors of Chinese residents (Ding et al., 2018; Wang and Zhou, 2020; Yang et al., 2021). In the development of LCCP, social media propaganda, low-carbon school education, and National Low-carbon Day activities are conducted as initiatives to foster the low-carbon atmosphere in society (Zhao et al., 2016; Ma et al., 2021). Therefore, LCCP cultivates residential behavioral habits by configuring social subjective norms.

Hypothesis 3: Low-carbon city pilots would establish subjective norms of residents to promote emissions mitigation behaviors.

Finally, perceived behavioral control refers to the degree of difficulty individuals feel about performing a particular behavior. Perceived behavioral control includes perceptions of facilitators or hindrances (such as convenience, economy, and time conditions), as well as perceptions of the degree of influence of these factors. Concretely, individual behaviors will be adapted when conditions such as spatial planning, transportation design, housing conditions, and public services are within local conditions (Zhang et al., 2020). When residents have experienced climate change impacts, or their local environment is under serious climate threat, they would formulate higher perceptions of climate change and will be more likely to implement low-carbon behaviors (Wang and Zhou, 2020). Pilot cities have also influenced residential perceived behavioral control through carbon inclusive policies, such as subsidizing green travel, developing public transportation, rewarding energy and water conservation, and establishing credit systems (Li et al., 2018). Therefore, LCCP tries to promote residents to implement low-carbon behaviors by improving positive factors of behavioral perceptions.

Hypothesis 4: Low-carbon city pilots would adjust the perceived behavioral control of residents to promote emissions mitigation behaviors.

## 3. Methodology and data

### 3.1. Residential energy-related emissions

This study focuses on carbon emissions resulting from residential energy consumption behaviors in cities. In order to obtain accurate data, residential household living energy consumption can provide a source for carbon emissions accounting. Zheng et al. (2011) and Zhao et al. (2012) classified residential energy consumption activities into four categories: electrical appliances, central heating, private transportation, and activities utilizing fuels. Miao (2017) calculated residential energy-related emissions for 216 cities in China from the perspective of private transportation and house-based energy consumption. Particularly, house-based energy consumption includes three major energy consumption sectors: (1) electricity consumption; (2) central heating; and (3) natural gas, liquefied petroleum gas, and coal gas uses. In this study, residential energy-related emissions due to household living energy consumption are defined as those resulting from residential electricity, central heating, residential gas, and private transportation, according to existing studies and available data. Through accounting above four categories of energy consumption and carbon emissions, residential energy-related emissions in cities are aggregated. Accounting methods are shown in Table 1.


(1) $$\begin{array}{l} \begin{array}{c} REE_{it}=CC_{it}+CE_{it}+CH_{it}+CCG_{it}+CLPG_{it} \end{array} \end{array}$$

**Table 1:** Accounting carbon emissions inventory.

	**Equations**	**Descriptions**	
Residential electricity	CE_*i*_ = E_*i*_×EF_*e, r*_	CE*_*i*_*	Household electricity emissions of city *i* (t_CO2_)
		E*_*i*_*	Household electricity consumption city *i* (MWh)
		EF*_*e, r*_*	Electricity emission factors *e* of regional grid *r* (t*_*CO*2_*/MWn)
Central heating	CH_*i*_ = S_*i*_×N × EF_*c*_	CH*_*i*_*	Household central heating emissions of city *i* (t_CO2_)
		S_i_	Household central heating area of city *i* (m^2^)
		N	Coal consumption per unit area of heating (*t_*coal*_*/m^2^)
		EF*_*c*_*	Standard coal emission factor (t*_*CO*2_*/t*_*coal*_*)
Residential gas	CCG_*i*_ = CG_*i*_×EF_*cg*_	CCG_*i*_	Household gas emissions of city *i* (t_CO2_)
	CLPG_*i*_ = LPG_*i*_×EF_*lpg*_	CLPG_*i*_	Household LPG emissions of city *i* (t_CO2_)
		CG_*i*_	Household gas consumption of city *i* (m^3^)
		LPG_*i*_	Household LPG consumption of city *i* (t*_*ipg*_*)
		EF_*cg*_	Carbon emission factor of gas (t_CO2_/m^3^)
		EF_*lpg*_	Carbon emission factor of LPG (t_CO2_/t*_*lpg*_*)
Private transportation	CC_*i*_ = Pcar_*i*_×AM × EF_*f*_×K	CC_*i*_	Private vehicle emissions in city *i* (t_CO2_)
		Pcar_*i*_	Private vehicle stock in city *i* (1,000 vehicles)
		AM	Average annual mileage (km/year)
		K	Fuel efficiency (L/100 km)
		EF_*f*_	Fuel emission factor (t_CO2_/L)

Residential energy-related emissions *(REE*_*it*_*)* are the sum of residential electricity, central heating, residential gas, and private transport. *i* and *t* are dummy variables for city and year, respectively.

### 3.2. Model setting

To compare changes in residential energy-related emissions after LCCP, the DID model is adopted to examine policy effects. The National Development and Reform Commission of China issued three batches of LCCP notifications in 2010, 2012, and 2017, respectively. Since the second batch of notifications was issued at the end of 2012, considering policy lag, the pilot time is set to 2013. Three batches of pilots included 6 provinces, 77 cities, and 4 counties in total. Considering that, some of the pilot areas are provinces, prefecture-level cities within the pilot provinces would be taken as pilot cities (Yao and Shen, 2021). Total 120 prefecture-level cities included in the pilots are considered as research subjects. Therefore, in this study, pilot cities are regarded as the treatment group and non-pilot cities as the control group. The policy effects of LCCP on residential energy-related emissions will be examined by setting up the DID model:


(2) $$\begin{array}{l} \begin{array}{c} REE_{it}=\alpha +\beta \times did_{it}+\overset{n}{\underset{j=1}{\sum }}\delta \times Control_{jit}+\nu_{t}+\gamma_{i}+\varepsilon_{it} \end{array} \end{array}$$

where *REC*_*it*_ is CO_2_ emissions due to residential energy consumption in cities; *did*_*it*_ is a dummy variable used to recognize pilot cities; *Control*_*jit*_ represents a series of control variables with j types; α, β, and δ are series of estimated regression coefficients; ν_*t*_, γ_*i*_, and ε_*it*_ represent year fixed effects, city fixed effects, and random error terms; *i* and *t* are dummy variables for city and year, respectively.

Control variables include (1) GDP, which is usually served as a reference to measure the economic development of cities; (2) population, which represents the population level of cities; (3) technology, which reflects the technological innovation capacity of cities; (4) finance, which measures the saving capacity of residents in the financial sector of cities; and (5) industrial structure, which measures the structural composition of local industrial development. With reference to the existing study, the above control variables are represented by GDP per capita, population density, ratio of science and technology expenditure in GDP, ratio of deposits in financial institutions in GDP, and ratio of secondary industry, respectively (Song et al., 2020; Yu and Zhang, 2021; Huo et al., 2022).

### 3.3. Data sources

All 281 prefecture-level cities in China from 2004 to 2020 were taken as research samples. Data on central heating for household living were obtained from the China Statistical Yearbook of Urban Construction. Private urban car ownership is obtained from the traffic data of statistical yearbooks of prefecture-level cities. The rest of the data were acquired from the China City Statistical Yearbook. As mentioned earlier, some of the data are ratio, and non-ratio type data have been logarized (Huo et al., 2022; Liu et al., 2022) here. Missing values were filled in using interpolation (Song et al., 2020; Jia et al., 2021). The descriptive statistics of variables are shown in Table 2.

**Table 2:** Descriptive statistics of variables.

	**Variable**	**Indicators**	**Observations**	**Mean**	**Standard deviation**	**Min**	**Max**
Interpreted variables	*lnREE*	Household living energy consumption generated carbon emissions	4,777	5.081	1.102	0.478	11.850
	*lnperTEE*	City total emissions related to HIC	4,776	6.340	1.088	2.603	9.400
Mediators	*Garbage*	Garbage disposal per capita	4,178	0.115	0.163	0.000	1.995
	*Graduate*	Higher education students/population	4,700	0.017	0.022	0.000	0.131
	*Perbus*	Buses owned per 1,000 people	4,777	0.312	0.614	0.003	11.500
Control variables	*lnPerGDP*	GDP per capita	4,777	10.320	0.808	4.595	15.680
	*lnPopden*	Population density	4,777	5.700	0.931	1.547	9.086
	*Technology*	Science and technology expenditure/GDP	4,775	0.002	0.003	0.000	0.063
	*Finance*	Deposits in financial institutions/GDP	4,777	1.467	1.114	0.245	24.800
	*Industry*	Secondary industry value added/GDP	4,777	47.100	11.280	9.000	90.970

## 4. Empirical results

### 4.1. Benchmark regression results

Using equation (2), benchmark regression results for the policy effects of LCCP can be obtained, which is shown in Table 3. As observed from column (1), the coefficient β representing LCCP is significantly negative. It indicates that LCCP would significantly decrease carbon emissions from residential energy consumption. Upon this, control variables were added to the regression analysis. Column (2) means that the regression result is still negative and significant at a higher level after adding the control variables. For further investigation of the policy effects of LCCP, this study replaces the dependent variable total energy-related consumption related to electricity, central heating, gas, and private transportation generated carbon emissions in cities (*TEE*). *TEE* is not just limited to household energy consumption but includes total electricity consumption, central heating, gas consumption, and private transportation in cities. In columns (3) and (4), it is found that the coefficients β are still significantly negative. Thus, these results above preliminarily confirm the theoretical Hypothesis 1, that LCCP would decrease carbon emissions from residential energy consumption.

**Table 3:** Benchmark regression results.

	**(1)**	**(2)**	**(3)**	**(4)**
	**REE**		**TEE**	
Pilot (treat)	−0.077^**^	−0.079^***^	−0.093^**^	−0.093^**^
	(0.033)	(0.030)	(0.042)	(0.039)
lnPerGDP		0.288^***^		0.361^***^
		(0.070)		(0.099)
lnPopden		−0.116^*^		0.039
		(0.064)		(0.119)
Technology		9.381^***^		7.090
		(3.007)		(4.636)
Finance		0.013^*^		0.048^***^
		(0.007)		(0.017)
Industry		0.003^*^		0.002
		(0.002)		(0.003)
Constant	5.098^***^	2.590^***^	6.361^***^	2.249^*^
	(0.007)	(0.841)	(0.009)	(1.236)
City fixed effect	Yes	Yes	Yes	Yes
Year fixed effect	Yes	Yes	Yes	Yes
Observations	4,777	4,775	4,776	4,774
*R^2^*	0.951	0.955	0.924	0.929

Standard errors in parentheses.

* *p* < 0.1,

** *p* < 0.05,

*** *p* < 0.01.

### 4.2. Parallel trend analysis

Satisfying the parallel trend test is a prerequisite for the DID model (Hong et al., 2021; Jia et al., 2021; Liu et al., 2022). Parallel trends can test that there is no obvious difference in the trend of residential energy-related emissions between pilot cities and non-pilot cities before the implementation of LCCP. This study will adopt the event study method for parallel trend testing (Jia et al., 2021).


$$\begin{array}{lll} REC_{i} & = & \alpha +\overset{6}{\underset{t=-6}{\sum }}\lambda_{t}\times D_{it}+\overset{n}{\underset{j=1}{\sum }}\delta \times Control_{jit}+\nu_{t}+\gamma_{i} \end{array}$$

(3) $$\begin{array}{l} +\varepsilon_{it} \end{array}$$

**Figure 1:**
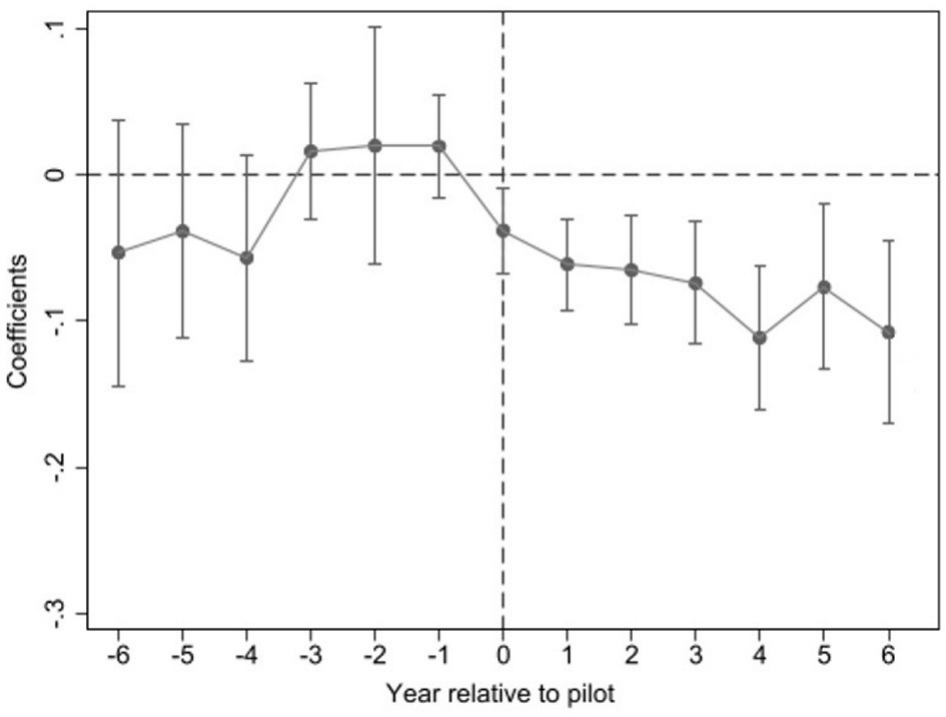
Parallel trend test.

In Equation (3), *D*_*it*_ denotes a dummy variable for whether city *i* is a pilot city in various years *t*. Other variables are the same as in equation (2). Equation (3) focuses on the coefficient λ_*t*_, which represents estimated coefficients of the policy effects of LCCP for pilot cities. Since the first batch of LCCP was implemented in 2010 and the time range of sample data is 2004–2020, this study picked 6 years before and after LCCP as the test time span. The pilot years are 2010, 2013, and 2017. In this study, the parallel trend test will be conducted for these three pilot periods together. The relative pilot years are used as the horizontal coordinates of Figure 1, with reference to the existing literature (Jia et al., 2021).

Figure 1 displays the results of the parallel trend test. Before the implementation of LCCP (left side of the dashed line), the policy effect is not significant and there is no common trend in coefficient values. However, after implementing LCCP (right side of the dashed line), the estimated coefficient values of λ_*t*_, are significantly negative and the absolute values show a gradual increasing trend. The solid dots indicate the estimated coefficients λ_*t*_ of equation (3), and the error bar are upper and lower 95% confidence intervals. Accordingly, this study considers that the sample passes the parallel trend test. The implementation of LCCP can achieve a significant and increasingly strong policy effect of decreasing residential energy-related emissions.

### 4.3. Robustness test

#### 4.3.1. Placebo test

Placebo tests can eliminate the disturbance of incidental events and thus demonstrate the robustness of regression results. In this study, the placebo test was conducted by randomly generating 281 sample cities and LCCP treatment groups. Randomly generated samples are re-estimated by using the DID model (Equation 2), which yields the estimated coefficients of simulated policy effects (Chen and Wang, 2022).

**Figure 2:**
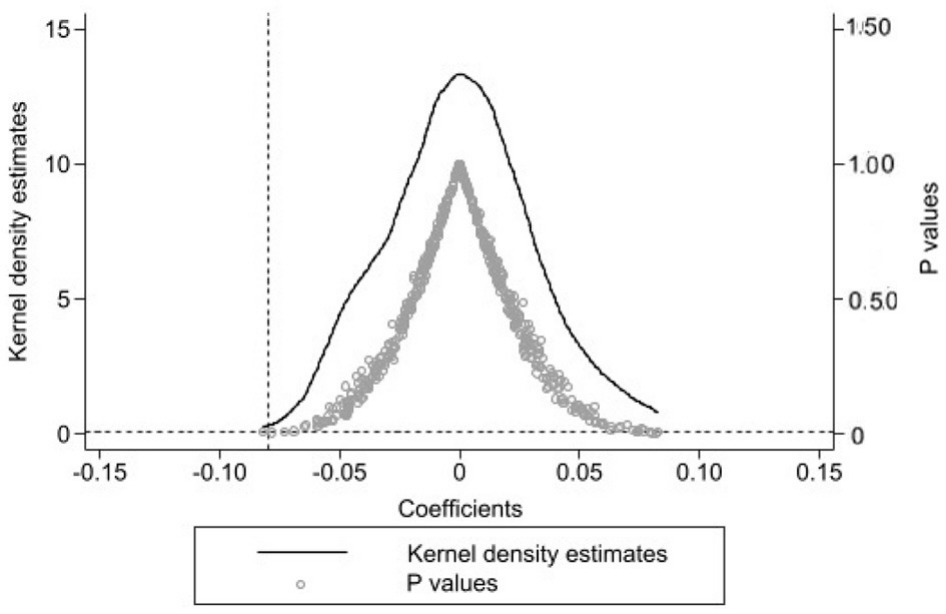
Placebo test.

By repeating the above placebo test 500 times, the distribution of estimated coefficients can be obtained, as shown in Figure 2. As observed in Figure 2, the estimated coefficient values significantly deviate from actual estimates (dashed line) and converge around the value of 0. It may also observe that the significance of the simulated regression is lower (*p* > 0.1). The results mentioned earlier indicate that benchmark regression results were not disturbed by other omitted factors (Jia et al., 2021). Thus, the estimated results of benchmark regression passed the placebo test and remained robust.

#### 4.3.2. Impact of multiple pilots

There are multiple pilot conditions in LCCP. In China, LCCPs have been issued in three batches in 2010, 2012, and 2017, respectively. In these three batches, there were 6 provinces that were granted pilots in the first two batches. However, after these provinces were granted pilots, the prefecture-level cities within pilot provinces have been included in the pilot list subsequently. This study set multiple pilot prefecture-level cities in benchmark regression according to the time when their provinces granted pilots. To reveal the policy effects of multiple pilots, this study reweights them with reference to existing studies (Liu et al., 2022). Specifically, the dummy variables for pilots will be reset. Cities that were granted once were assigned the value of 1, and cities that were granted twice at provincial and prefectural levels were assigned the value of 2.

The results of multiple pilots are presented in Table 4. Column (1) indicates that the estimated coefficient remains significant under the multiple pilot policies and the value increases over benchmark regression. It suggests that cities granted pilot eligibility twice have stronger incentives to decrease residential energy-related emissions. Simultaneously, it might also explain why the second and third batches of LCCP mainly chose cities.

**Table 4:** Robustness tests.

	**(1)**	**(2)**	**(3)**
	**Multiple pilots**	**ETP**	**Policy lag**
Pilot (treat)	−0.098^***^	−0.077^***^	−0.090^***^
	(0.028)	(0.029)	(0.027)
ETP		−0.017	
		(0.054)	
Control variables	Yes	Yes	Yes
City fixed effect	Yes	Yes	Yes
Year fixed effect	Yes	Yes	Yes
Observations	4,775	4,775	4,494
*R^2^*	0.956	0.955	0.960

Standard errors in parentheses.

^*^p < 0.1, ^**^p < 0.05,

*** *p* < 0.01.

#### 4.3.3. Impact of other pilot policies

China has launched a variety of emissions mitigation policies aiming to achieve carbon peaking and carbon neutrality targets. Two of these policies, LCCP and the emissions trading pilot (ETP), are the most representative. During the implementation of ETP, it is also exploring to include residents in certified emissions mitigation trading (Zhao et al., 2016; An et al., 2021). Beijing and Guangzhou have started to offer trading services for residents. Although the trading volume is relatively small, it will likely influence the policy effects of LCCP.

There may be interferences between policies, which in turn affect the measurement of policy effects. Such cases need to include related policies in the scope of the investigation to observe whether the original policy effect would be affected. First, the estimated coefficients are insignificant, implying that the original policy effect does not exist. Second, the estimated coefficients become smaller but still significant. It means that the original policy effect is overestimated. Third, the estimated coefficients become large and significant. At this point, the original policy effect may be underestimated (Song et al., 2020; Liu et al., 2022).

After adding ETP, the result is presented in Table 4. Column (2) shows that the original policy effect is still significantly negative compared to benchmark regression. However, a slight decline in the estimated coefficient values can be found, indicating a certain degree of overestimation of the original policy effect. In addition, it is also shown that the coefficient value of ETP is negative, indicating a negative, but not significant, policy effect of ETP.

#### 4.3.4. Impact of policy lag

Lag in policy effects is common. There are a series of processes to generate policy effects, including agenda building, issuing notifications, implementing fulfillment, and finally a period of time before policy effects are generated. This study sets the implementation time in 2013 for the second batch of LCCP released in 2012 for this reason. In this study, a robustness test is conducted for the lag of policy effects by referencing previous studies (Yu and Zhang, 2021; Chen and Wang, 2022). Three batches of LCCP were notified in 2010, 2012, and 2017, and all three batches will be lagged by 1 period in the policy lag test, results shown in Table 4.

Column (2) implies that the regression results are negative and significant. It indicates that there is the policy lag in LCCP. By comparing with benchmark regression, the coefficient in column (2) results is bigger. This means that there is the policy lag while the effect becomes increasingly stronger. This has long-term implications for residential energy consumption decrease under low carbon city construction.

## 5. Further analysis

### 5.1. Influencing mechanism analysis

Both benchmark regression and robustness tests have been able to show that LCCP can decrease carbon emissions from residential energy consumption. How does the policy effect work, in other words, by what mechanisms would LCCP influence residential energy consumption and carbon emissions? Based on TPB, residential low-carbon behaviors are influenced by perceptions. Therefore, this study proposes Hypotheses 2–4 in 2.2. These three hypotheses explore the influencing mechanisms of LCCP on residential energy-related emissions in terms of behavioral attitudes, subjective norms, and perceived behavioral control, respectively. In order to test the above hypotheses of influence mechanisms, this study adopts the mediating model to validate (Chen et al., 2021; Liu et al., 2022).


(4) $$\begin{array}{l} \begin{array}{c} M_{it}=\alpha +\varphi \times did_{it}+\overset{n}{\underset{j=1}{\sum }}\delta \times Control_{jit}+\nu_{t}+\gamma_{i}+\varepsilon_{it} \end{array} \end{array}$$

First, LCCP may strengthen the behavioral attitudes of residents to promote emissions mitigation behaviors. When residents have positive or negative affective tendencies toward low-carbon behaviors, they are more likely to display emissions mitigation behaviors or not. For example, those who show supportive attitudes toward low-carbon consumption intentions are more likely to purchase environmentally friendly products and support consumption-side emissions mitigation policies (Yang et al., 2014; Ding et al., 2018; Tian et al., 2022). To validate the mechanism, this study used the amount of per capita garbage harmlessly treated in cities to measure residential behavioral attitudes. The reason for this is that domestic garbage harmlessly treated represents the willingness of residents to engage in environmentally friendly behavior (Ek and Miliute-Plepiene, 2018). If residents are able to actively participate in domestic garbage harmlessly treated, then it indicates that they have positive attitudes toward environmental improvement behaviors. At the same time, domestic garbage harmlessly treated can also decrease a part of GHG from residential non-energy consumption (Chen et al., 2020). In Table 5, column (1) demonstrates that LCCP increases the amount of per capita domestic garbage harmlessly treated with a strong significance.

**Table 5:** Mechanism tests.

	**(1)**	**(2)**	**(3)**
	**Garbage**	**Graduate**	**Bus**
Pilot (treat)	0.041^***^	0.002^**^	0.059^***^
	(0.012)	(0.001)	(0.022)
Control variables	Yes	Yes	Yes
City fixed effect	Yes	Yes	Yes
Year fixed effect	Yes	Yes	Yes
Observations	4176	4698	4775
*R^2^*	0.849	0.941	0.904

Standard errors in parentheses. ^*^p < 0.1,

** *p* < 0.05,

*** *p* < 0.01.

Second, LCCP would establish subjective norms for residents to promote mitigation behaviors. Subjective norms can form external pressures on residents. These pressures may stem from many subjective factors such as psychological distance, emotional characteristics, political orientation, and education level. Subjective norms play a crucial role in residential energy consumption mitigation behaviors due to the influence of traditional collectivist values culture in China (Webb et al., 2013; Mancha and Yoder, 2015; Chen, 2016). In China, education is the largest subjective factor influencing the behaviors of residents (Wang and Zhou, 2020). Therefore, the ratio of higher education to the total urban population in cities was chosen to validate the mechanism of subjective norms, as shown in Table 5. As demonstrated in column (1), LCCP significantly increases the ratio of higher education in the total urban population.

Third, LCCP could adjust the perceived behavioral control of residents to promote emissions mitigation behaviors. When residents perceive the degree of difficulty in implementing low-carbon behaviors, such perceptions can facilitate or hinder the occurrence of behaviors. In particular, spatial planning, transportation design, housing conditions, public services, and other conditions in cities could influence residential energy consumption and emissions mitigation behaviors (Zhang et al., 2020). Therefore, many pilot cities have adopted initiatives such as strict building energy efficiency standards, subsidizing the purchase of new energy vehicles, improving public transportation services, and rewarding energy and water saving behaviors to influence residential behavior perceptions (Liu and Qin, 2016; Zhao et al., 2016; Li et al., 2018). To validate the influence mechanism of perceived behavioral control, this study uses the number of buses owned per 1,000 people in cities. This is because buses are one of the low-carbon options for travel for residents in cities. Moreover, the number of buses could also reflect cities' efforts to mitigate emissions in the transportation sector. Column (3) demonstrated that LCCP increased bus ownership per 1,000 people and was significant.

Through the above mechanism analysis, it is clear that LCCP could strengthen behavioral attitudes, establish subjective norms, and adjust perceived behavioral control to shape residential low-carbon perceptions and subsequently drive emissions mitigation behaviors. Such results confirm that Hypotheses 2–4.

### 5.2. Heterogeneity analysis

The vastness of China gives birth to distinct differences between cities in terms of geographic conditions, economic development, and institutional arrangements. The benchmark regression robustness test does not take into account these differences in location and size. For a deeper understanding of the differences in policy effects of LCCP across cities, this study examines the heterogeneity of geographic location and city size.

Cities located in various regions have differences in energy structure, economic development, emissions levels, and policy implementation standards. In this study, Chinese cities are divided into eastern, central, and western regions, as shown in Table 6. Columns (1–3) demonstrate the differences in policy effects of LCCP among these three regions. The estimated coefficients for eastern and central regions are significantly negative. It indicates that LCCP within eastern and central regions can help to decrease residential energy-related emissions. It is possible that due to the high level of economic development, concentration of educational resources, and availability of infrastructure in eastern and central regions. These contribute to the low-carbon perception of residents, who are willing to implement energy saving and emissions mitigation behaviors (Shen et al., 2021; Yang et al., 2021). Column (3) demonstrates that the estimated coefficient for the western region, although also negative, is not significant. Economic growth is still a priority in the western region, and insufficient management of energy consumption and lack of low-carbon perceptions in society, which in turn leads to the ineffectiveness of carbon emissions mitigation among residents. Thus, the implementation of LCCP in the western region is more difficult and policy effects are not effective.

**Table 6:** Heterogeneity analysis of cities.

	**(1)**	**(2)**	**(3)**	**(4)**	**(5)**
	**Eastern**	**Central**	**Western**	**Bigger**	**Smaller**
Pilot (treat)	−0.075^**^	−0.087^*^	−0.034	−0.116^***^	−0.069^*^
	(0.037)	(0.051)	(0.081)	(0.043)	(0.039)
Control variables	Yes	Yes	Yes	Yes	Yes
City fixed effect	Yes	Yes	Yes	Yes	Yes
Year fixed effect	Yes	Yes	Yes	Yes	Yes
Observations	1,649	2,091	1,035	2,193	2,582
*R^2^*	0.964	0.947	0.945	0.966	0.950

Standard errors in parentheses.

* *p* < 0.1,

** *p* < 0.05,

*** *p* < 0.01.

Bigger cities, due to their economic development, massive population, and dense transportation, simultaneously generate the “big city disease,” such as environmental pollution, energy consumption, and resource scarcity (Cheng et al., 2019). Insufficient development, inadequate infrastructure, and environmental problems in smaller cities may also have an impact on policy effects. According to the Notice on Adjusting the Size of Cities issued by the State Council of China in 2014, this study divides pilot cities into two groups: megacities and large (bigger) cities, and medium and small (smaller) cities, as shown in Table 6. Columns (4) and (5) show the regression results in these two groups of cities. The coefficient estimates are all significant, which indicates that LCCP could achieve residential energy-related emissions mitigation in both bigger cities and smaller cities. By comparing the results in these two columns, it can be observed that the estimated coefficients are larger and more significant for bigger cities. These suggest that LCCP could be more helpful in mitigating residential energy-related emissions in bigger cities. The possible reason for this is that residents in bigger cities face more climate threats, such as extreme heat, health loss, and energy shortage. Therefore, they are more concerned about environmental issues and have stronger low-carbon perceptions, which in turn help to drive energy-saving behaviors (Huo et al., 2022). In contrast, smaller cities are still in the development stage and have relatively modest emissions levels, thus residential low-carbon perceptions are not as strong as bigger cities. Meanwhile, the infrastructure in smaller cities is insufficient, and the cost of emissions mitigation for residents is higher than inputs, resulting in fewer residential low-carbon perceptions and energy-saving behaviors.

## 6. Conclusion and policy implications

This study focused on residential low-carbon perceptions and energy consumption behaviors in the policy context of LCCP. The examination of residential energy-related emissions changes under LCCP provided a new perspective to evaluate policy effects. Introducing TPB increased the explanatory power for analyzing how LCCP affected residential low-carbon perceptions, subsequently driving energy-saving behaviors and decreasing carbon emissions. Further analysis validated the influence mechanisms and heterogeneity affecting the policy effects of LCCP.

The main findings are as follows: First, LCCP would yield the policy effect of decreasing residential energy-related emissions. Results passed the robustness tests of the placebo, multiple policies, other policies, and policy lag. In particular, multiple pilot eligibility and policy lag could decrease greater residential energy-related emissions, while the policy effect of LCCP with ETP may be slightly overestimated. Second, residential energy-related emissions are accounted for by combining existing studies and available data. The emissions inventory scope includes residential electricity, central heating, residential gas and private transportation. Third, based on TPB theory, the influencing mechanism analysis validated that LCCP would strengthen behavioral attitudes, establish subjective norms, and adjust perceived behavioral control. The causal chain that LCCP shaped residential low-carbon perceptions, subsequently driving energy-saving behaviors and decreasing carbon emissions, was founded. Finally, the policy effects of LCCP are heterogeneous across geographic locations and city sizes. Policy effects were more significant in eastern and central regions, but not in western regions. Although LCCP would decrease residential energy-related emissions in both bigger cities and smaller cities, the policy effect was stronger in bigger cities.

Based on the previous findings, this study proposes several policy recommendations that are helpful to decrease residential energy-related emissions. (1) Energy saving and emissions mitigation policies reinforce concerns about residential low-carbon lifestyles. Improving residential living quality and forming a low-carbon atmosphere are the target of policy design. Direct or indirect behaviors of residents will lead to energy consumption and carbon emissions. In the process of constructing low-carbon cities, it is essential to take residential energy-related emissions mitigation as a priority in policy implementation, improve institutional systems, explore feasible approaches, and increase assessment weights, in order to build low-carbon and high-quality living environments in cities. (2) Enhancing residential low-carbon perceptions is the driving force for taking emissions mitigation behaviors. Attitudes toward low-carbon behaviors, social pressure from subjective norms, and the difficulty of implementing low-carbon behaviors jointly influence the shape of residential low-carbon perceptions. For promoting emissions mitigation in residential lifestyles and energy consumption behaviors, shaping residential behavioral attitudes, subjective norms, and perceived behavioral control should be incorporated into the policy scope, by reinforcing residential low-carbon perceptions and subsequently driving the occurrence of low-carbon behaviors. (3) Cities promoting energy saving and emissions mitigation among residents should combine local characteristics. Differences in geographic location and city size make cities have different economic development, energy structures, social perceptions, emissions levels, and policy capabilities. When constructing low-carbon cities and decreasing residential energy-related emissions, these differences will affect the policy effects. Cities should adapt their policy implementation according to local characteristics to avoid the phenomenon of “going with the flow” and “one size fits all,” achieving low-carbon and high-quality development.

The policy effects and influencing mechanisms of LCCP on residential energy-related emissions have been validated. Nevertheless, there are multiple possible emissions pathways in residential lifestyle and energy consumption, which might be influenced by additional factors. Since LCCP has been implemented for a relatively short time and data were limited. Therefore, it is necessary to further expand the scope of collecting residential energy-related emissions, find out the potential influencing factors, and track the policy effects in long-term for future researches.

## Data availability statement

The raw data supporting the conclusions of this article will be made available by the authors, without undue reservation.

## Author contributions

HX: supervision and review. XL: conceptualization, methodology, data, writing, and editing. All authors contributed to the article and approved the submitted version.
